# COVID-19 Vaccinations: Summary Guidance for Cancer Patients in 28 Languages: Breaking Barriers to Cancer Patient Information

**DOI:** 10.2174/1574887116666211028145848

**Published:** 2022-05-10

**Authors:** Davide Mauri, Konstantinos Kamposioras, Lampriani Tsali, Mario Dambrosio, Berardino De Bari, Nadia Hindi, Carl Salembier, Joanna Nixon, Tzachanis Dimitrios, Flippo Alongi, Hassan Hameed, Antonios Valachis, Konstantinos Papadimitriou, Stefanie Corradini, Lazar Popovic, Jindrich Kopecky, Andres Rodriguez, Katarina Antunac, Junlin Yi, Jozsef Lovey, Primoz Strojan, Haytham Saraireh, Ranveig Røtterud, Marzanna Chojnacka, Santa Cruz Olalla, Natalia Chilingirova, Ramon Andrade De Mello, Giovanna Araujo Amaral, Farsid Arbabi, Radu Vidra, Erjeta Rapushi, Dan Takeuchi, Chirstos Christopoulos, Irina Ivanova, Igor Djan, Branka Petricevic, Francesco Cellini, Iglika Mihaylova, Natalija Dedic Plavetic, Cvetka Grašič Kuhar, Elena Takeuchi, Pantelis Kountourakis, Panagiotis Ntellas, Ioanna Gazouli, Stefania Gkoura, Salih Yuce, Özlem ER, Chait Yasmina, Gireesh Kumaran, Orges Spahiu, Aasim Yusuf, Paulina Gono, Kathi Apostolidis, Maria Tolia

**Affiliations:** 1 Department Medical Oncology, University Hospital of Ioannina, Ioannina, Greece;; 2 Department. Medical Oncology, The Christie NHS Foundation Trust, Manchester, UK;; 3 PACMeR Evidence Based Medicine, Athens, Greece;; 4 Department. Medical Oncology, Clinica San Carlo, Paderno Dugnano, Milano Italy;; 5 Service Radio-Oncologie Neuchâtel Hôpital Network, La Chaux-de-Fonds, Switzerland;; 6 Department. Medical Oncology, Fundación Jimenez Díaz University Hospital, Madrid, Spain;; 7 Radiation Oncology Department, Europe Hospitals, Brussels, Belgium;; 8 Beatson West of Scotland Cancer Center, Strathclyde University, Glasgow, UK;; 9 UCSD Blood and Marrow Transplant Program, UCSD/Moores Cancer Center, La Jolla, CA, USA;; 10 Advanced Radiation Oncology Department, IRCCS Sacro Cuore Don Calabria, Negrar-Verona,University of Brescia, Verona, Italy;; 11 The Christie NHS Foundation Trust, Manchester UK, The Christie's Hospital, Manchester, UK;; 12 Department Medical Oncology, Faculty of Medicine and Health, Örebro University, Örebro, Antonios, Sweden;; 13 Department of Medical Oncology, University Hospital of Antwerp, Antwerp, Belgium;; 14 Department Radiation Oncology, University Hospital, LMU Munich, Germany;; 15 Department Medical Oncology,Oncology Institute of Vojvodina, University of Novi Sad, Serbia;; 16 Department Oncology and Radiotherapy, Charles University, Hradec Králové, Prague, Czech Republic;; 17 Medical Oncology, Instituto Alexander Fleming in Buenos Aires, Buenos Aires, Argentina;; 18 University Hospital for Tumors, Sestre Milosrdnice University Hospital Center, Zagreb, Croatia;; 19 Department Radiation Oncology, National Cancer Center/Cancer Hospital, Chinese Academy of Medical Sciences, Peking Union Medical College, Beijing, China;; 20 National Institute of Oncology, Semmelweis University, Budapest, Hungary;; 21 Department Radiation Oncology, Institute of Oncology, Ljubljana, Slovenia;; 22 Department Radiation Oncology, Royal Medical Service, Amman, Jordan;; 23 Cell Biologist, CEO Bladder Cancer, Oslo, Norway;; 24 Department of Oncology and Radiotherapy, Maria Skłodowska-Curie National Research Institute of Oncology, Warsaw, Poland;; 25 Service de Radio-Oncologie, Neuchâtel Hôpital Network, La Chaux-de-Fonds, Switzerland;; 26 Department Medical Oncology, Center of Excellence, Heart and Brain Hospital Pleven, Medical University Pleven, Bulgaria;; 27 Department Medical Oncology Escola Paulista de Medicina, Federal University of São Paulo, Brazil, Department Biomedical Sciences,and Medicine, University of Algarve, Faro, Portugal;; 28 Department Medical Oncology, Escola Paulista de Medicina, Federal University of São Paulo, Brazil;; 29 Department Medical Oncology, Rooshana Cancer Center, Tehran, Iran;; 30 Medical Oncology, Regional Institute of Gastroenterology and Hepatology “Prof. Dr. O. Fodor”, Cluj-Napoca, Romania;; 31 Department Medical Oncology, Berat Regional Hospital, Berat, Albania;; 32 Matsudo City General Hospital, Chiba, Japan;; 33 Service de Radiothérapie Oncologique, GHT Grand Paris Nord-Est. G.H.I Le Raincy –Montfermeil, France;; 34 Murmansk Branch of All-Russian Association of Cancer Patients “Zdravstvuy”, NGO Center of Initiatives Support, Murmansk, Russia;; 35 Department for Radiosurgery and SBRT, Clinical center of Serbia, Belgrade, Serbia;; 36 Department of Medical Oncology and Hematology,Wilhelminenspital, Vienna, Austria;; 37 Radioterapia Oncologica-Fondazione Policlinico A. Gemelli, IRCCS –Rome, Italy;; 38 Department of Radiotherapy,Specialized Hospital for Active Treatment in Oncology “Plovdivsko pole”, Sofia, Bulgaria;; 39 Department of Oncology, University Hospital Centre Zagreb, School of Medicine University of Zagreb, Zagreb, Croatia;; 40 Department Medical Oncology, Institute of Oncology, Ljubljana, Slovenia;; 41 The Christie NHS Foundation Trust, Manchester, UK;; 42 Department Medical Oncology,Bank of Cyprus Oncology Centre, Nicosia, Cyprus;; 43 Chairmen of the Board-Young Saving Association, Turkey;; 44 Acıbadem University, Faculty of Medicine, Turkey;; 45 Service de Haematologie, GHT Grand Paris Nord-Est. G.H.I Le Raincy –Montfermeil, France GHT Grand Paris Nord-Est, G.H.I Le Raincy –Montfermeil, France;; 46 Medical Oncology, Auckland Regional Cancer and Blood Service, Auckland, New Zealand;; 47 Radiation Therapy Unit, University Hospital Center “Mother Teresa”, Tirana, Albania;; 48 Gastroenterology, Acting CEO Shaukat Khanum Memorial Cancer Hospital & Research Centre, Lahore and Peshawar Pakistan;; 49 ECPC Partneships and Communication Officer;; 50 ECPC President;; 51 Department. Radiation Oncology, University Hospital of Heraklion, Crete, Greece

**Keywords:** Population, vaccination, cancer patients, languages, global, guidance

## Abstract

***Background*:** Covid-19 vaccination has started in the majority of the countries at the global level. Cancer patients are at high risk for infection, serious illness, and death from COVID-19 and need vaccination guidance and support.

Guidance availability in the English language only is a major limit for recommendations’ delivery and their application in the world’s population and generates information inequalities across the different populations.

***Methods*:** Most of the available COVID-19 vaccination guidance for cancer patients was screened and scrutinized by the European Cancer Patients Coalition (ECPC) and an international oncology panel of 52 physicians from 33 countries.

***Results*:** A summary guidance was developed and provided in 28 languages in order to reach more than 70 percent of the global population.

***Conclusion*:** Language barrier and e-guidance availability in the native language are the most important barriers when communicating with patients. E-guidance availability in various native languages should be considered a major priority by international medical and health organizations that are communicating with patients at the global level.

## 1 BACKGROUND

Covid-19 vaccination has started in the majority of the countries. Nonetheless, the type of vaccine used and the vaccination protocols vary across the different countries. Since cancer patients are at higher risk of Covid-19 infection and sequels, they experience anxiety and need vaccination guidance and support.

In response to this need, we plan to summarize in plain terms and 28 languages the guidance for vaccination for cancer patients.

## 2 METHODS

The international oncology panel of 52 physicians from 33 countries, with the cooperation of the European Cancer Patients Coalition (ECPC), which had already published the summary of international recommendations for patients with cancer during the COVID-19 pandemic in 23 languages in Lancet Oncology (June 2020) [1], collected and summarized the COVID-19 vaccination guidance for cancer patients. Each nation’s representative from 33 different countries was asked to answer the status of the COVID-19 vaccination national guidance for cancer patients by the end of January 2021. The answers given were last updated at the end of March 2021. The main Oncology societies, namely ASCO, ESMO, ASTRO, and ESTRO, were also screened for guidance vaccination delivery.

## 3 RESULTS

Summary vaccination guidance for cancer patients was developed by guidance from 33 countries and 5 international institutions/organizations. [Table **1**. Appendix -page 81].

Guidance was then translated into 28 languages by each nation’s representative of the group and thereafter was delivered to the global cancer patients through the European Cancer Patients Coalition (ECPC) website. All working group participants cooperated on a voluntary basis. No financial contribution was required, and there is no conflict of interest. Covid-19 vaccination has been launched in all participating countries. [Supplementary Material Table **1**. Appendix -page 81].

## 4 DISCUSSION

Guidance availability in the English language only is a major limit for recommendations’ delivery and their application in the world’s population [1, 2].

**Fig. (1):**
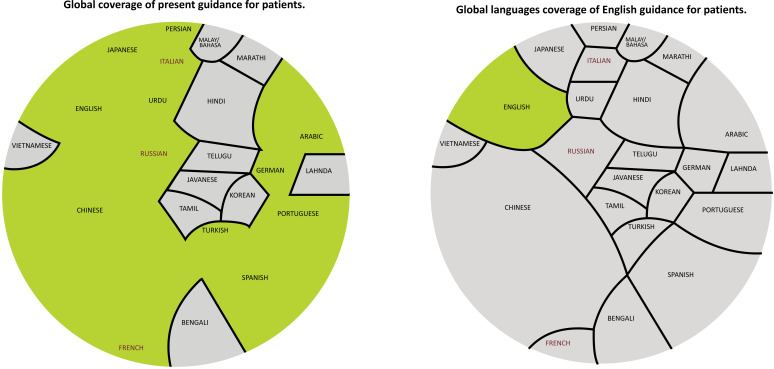
Demographics of the 23 most spoken languages as a native tongue (with at least fifty million first language speakers).

Indeed, in the world today, there are 7.2 billion people who speak more than 7,102 languages. Only 23 languages are spoken by at least 50 million people. More than half of the world’s population is native speakers of at least one of these 23 languages. English native speakers represent only 8% of the populations that speak the 23 most spoken languages [2] (Fig. **1**).

We thereafter provided our summary guidance in 28 languages in order to reach more than 70% of the global population. (Albanian, Arabic, Bulgarian, Catalan, Chinese, Croatian, Czech, Nederlands, English, French, German, Greek, Hungarian, Italian, Japanese, Latam, Norwegian, Persian-Farsi, Polish, Portuguese, Romanian, Russian, Serbian, Slovenian, Spanish, Swedish, Turkish, Urdu) (Fig. **1**).

Nonetheless, for a non-English speaking cancer patient, it is particularly difficult to surf a website in the English language in order to find guidance in his own native language. This generates information inequalities across the different populations.

Since the internet is a global phenomenon available in all languages, the only way to avoid disparities in information at the global level is to create easy access to multilanguage information. This can be reached by enabling patients to search guidance through google and internet searches with the use of keywords directly in their own native language.

We thereafter aim to provide searchable translations directly by titles and keywords in any language and a free-access setting.

## CONCLUSION

If the information is easily searchable on the web, delivered in plain terms, and available in a variety of native languages around the world, it can be made accessible to the global patient population without causing inequalities. Our initiative will hopefully assist cancer patients from different countries to have equal and easy access to information on covid immunization.

## CONSENT FOR PUBLICATION

Not applicable.

## FUNDING

None.

## SUMMARY GUIDANCE IN 28 LANGUAGES (APPENDIX)

- Summary guidance for Covid-19 vaccination for cancer patients with references (English) (appendix, page 2).

- Udhëzime përmbledhëse për vaksinimin kundër COVID-19 për pacientët me kancer (SHQIP) (Shtojca, faqe 6).

- Обобщени препоръки за ваксинация при онкологични пациенти-Covid-19 (Български език) (приложение, страница 11)

- Guia resumida de vacunació Covid-19 dels pacients amb càncer (en llengua catalana) (apèndix, pàgina 14)

- Sažetak smjernica za cijepljenje protiv COVID-19 za bolesnike oboljele od zloćudnih bolesti (Hrvatski) (dodatak, stranica 19)

- Souhrnné pokyny pro očkování pacientů s nádorovým onemocněním COVID 19 (čeština) (příloha, strana 22)

- Beknopte richtlijnen voor Covid-19 vaccinatie van kankerpatiënten (Nederlands) (bijlage, pagina 25)

- Summary guidance for Covid-19 vaccination for cancer patients. (English, without references) (appendix, page 28)

- COVID 19: Zusammenfassender Leitfaden zur Impfung für Krebspatienten (in Deutscher sprache) (Anhang, Seite 31)

- Οδηγίες για τον εμβολιασμό Covid-19 για ογκολογικούς ασθενείς (Ελληνικά) (παράρτημα, σελίδα 33)

- Összefoglaló útmutatás daganatos betegek számára a COVID-19 elleni oltásról (Magyar) (melléklet, 36. oldal)

- Indicazioni per la vaccinazione Covid-19 per i pazienti oncologici (Italiano) (appendice, pagina 38)

- Podsumowanie zaleceń dotyczących szczepień przeciw COVID-19 dla pacjentów onkologicznych. (Polski) (załącznik, strona 41)

- Uputstvo za vakcinaciju protiv Covid19 za pacijente obolele od malignih bolesti (Srpski) (dodatak, strana 43)

- Povzetek navodil za cepljenje bolnikov z rakom (Slovenščina)-Covid 19 (priloga, stran 45)

- Recomendaciones de vacunación Covid-19 para pacientes oncológicos (Español) (apéndice, pág. 48)

- Vägledning kring vaccination mot COVID-19 hos cancerpatienter (Svenska) (bilaga, sida 51)

- Ghid de vaccinare Covid-19 a pacienților oncologici. (Română) (Anexă, pagina 54)

- Veiledning for Covid-19-vaksinering av kreftpasienter. (Norsk) (vedlegg, side 57)

- Orientações resumidas para a vacinação de Covid-19 para pacientes com câncer (Portugues) (apêndice, página 60)

- Guía de vacunación COVID-19 para pacientes oncológicos (Latam) (apêndice, página 63)

- Synthèse de recommandations concernant la vaccination COVID-19, chez les patients cancéreux (Français) (annexe, page 70)

- Краткое руководство по вакцинации Covid-19 для онкологических больных, коренных носителей русского языка. (приложение, стр. 72)

- Kanser hastalarının Covid-19’a karşı aşılanmaları için özet kılavuz. (Türkçe) (ek, sayfa 78)

- Global coverage of present guidance for patients.

- Global languages coverage of English guidance for patients.
